# SGLT2 Inhibitors and Cardiovascular Outcomes in Patients With Fabry Disease: Observations From a Federated Research Network

**DOI:** 10.1111/dom.71324

**Published:** 2026-09-08

**Authors:** Leandra Serio, Michele Rossi, Amir Askarinejad, Enrico Tartaglia, Tommaso Bucci, Enrica Marrone, Dinesh K. Kalra, Claudio Ferri, Gregory Y. H. Lip

**Affiliations:** ^1^ Institute of Life Course and Medical Sciences University of Liverpool Liverpool UK; ^2^ Department of Life, Health and Environmental Sciences University of L'Aquila L'Aquila Italy; ^3^ Internal Medicine and Nephrology Division, ASL1 Avezzano‐Sulmona‐L'Aquila, San Salvatore Hospital L'Aquila Italy; ^4^ Department of Biomedical, Metabolic and Neural Sciences University of Modena and Reggio Emilia, Policlinico di Modena Modena Italy; ^5^ Clinical and Experimental Medicine PhD Program University of Modena and Reggio Emilia Modena Italy; ^6^ Internal, Vascular and Emergency Medicine—Stroke Unit University of Perugia Perugia Italy; ^7^ Infiltrative Cardiomyopathy Program, Division of Cardiology University of Louisville Louisville Kentucky USA; ^8^ Danish Center for Health Services, Department of Clinical Medicine Aalborg University Aalborg Denmark; ^9^ Department of Cardiology, Lipidology and Internal Medicine With Intensive Coronary Care Unit Medical University of Bialystok Bialystok Poland

**Keywords:** Anderson–Fabry disease, chronic kidney disease, heart failure, hypertrophic cardiomyopathy, SGLT‐2 inhibitors

## Abstract

**Background and Aim:**

Fabry disease (FD) is frequently complicated by renal impairment and cardiovascular events; however, evidence on sodium–glucose cotransporter‐2 inhibitors (SGLT2i) in this population is scarce. Accordingly, we assessed the association between SGLT2i exposure and cardiovascular outcomes in adults with FD.

**Methods:**

We conducted a retrospective cohort study using the TriNetX Global Collaborative Network, including adults with Fabry disease and eGFR ≥ 20 mL/min/1.73 m^2^, excluding Type 1 diabetes. Patients were stratified by SGLT2 inhibitor exposure. The primary outcome was a composite of all‐cause death, acute myocardial infarction and acute or acute‐on‐chronic heart failure at 2 and 5 years; secondary outcomes included individual components and progression to advanced kidney disease. Patients with prior non‐fatal outcomes were excluded from the respective analyses. Propensity score matching (1:1) and Cox proportional hazards models were used. Sensitivity analyses included alternative follow‐up periods, restriction to Fabry disease diagnosed from 2013 onwards and a stricter Fabry disease definition requiring ≥ 2 diagnoses; subgroup analyses were performed by sex and age.

**Results:**

Among 14 940 FD patients, 1028 (6.9%) had recorded exposure to SGLT2 inhibitors (mean age: 65.5 ± 12.0 years; 53.0% male), who were older and had a higher comorbidity burden and worse baseline renal profile than non‐exposed patients. After PSM, 732 patients per group (exposed and non‐exposed patients) were available for analysis. At 2 years, SGLT2i exposure was associated with a lower risk of the composite outcome (HR 0.61; 95% CI 0.38–0.97) and all‐cause death (HR 0.56; 95% CI 0.33–0.95). At 5 years, results were consistent for the composite outcome (HR 0.56; 95% CI 0.39–0.79) and all‐cause death (HR 0.62; 95% CI 0.42–0.93), with reduced myocardial infarction risk (HR 0.53; 95% CI 0.30–0.90).

**Conclusions:**

In a large real‐world FD cohort, SGLT2i exposure was associated with lower cardiovascular risk and mortality, supporting further prospective investigation.

## 1 Introduction

Anderson–Fabry disease (FD) is a rare X‐linked lysosomal storage disorder caused by pathogenic variants in the *GLA gene*, which encodes *α*‐galactosidase A, resulting in partial or complete deficiency of the enzyme. The reduced enzymatic activity leads to progressive intracellular accumulation of glycosphingolipids, particularly globotriaosylceramide (Gb3; also known as GL‐3) and related derivatives, ultimately promoting cellular dysfunction, inflammation and organ fibrosis [1].

FD is a systemic disorder with marked phenotypic heterogeneity, ranging from the classic early‐onset form to later‐onset variants and may involve multiple organs, including the nervous system, skin, eyes, gastrointestinal tract, kidneys, heart and cerebrovascular system [2, 3]. Among these manifestations, cardiac and renal involvement are major determinants of disease progression and prognosis. Cardiac disease in FD typically presents as a non‐ischemic hypertrophic cardiomyopathy, initially associated with diastolic dysfunction and progressive myocardial remodelling, and later evolving towards replacement fibrosis [4], arrhythmias and heart failure (HF) [1, 5, 6]. In parallel, Fabry nephropathy is characterised by glomerular and tubulo‐interstitial damage, leading to a progressive decline in renal function that may culminate in advanced chronic kidney disease [2]. The currently available disease‐specific therapies, particularly enzyme replacement therapy [5, 7], have modified the natural history of FD; however, a substantial proportion of patients continue to experience progressive cardiorenal involvement despite treatment.

Sodium–glucose co‐transporter 2 inhibitors (SGLT2i) have shown a clinically relevant impact on HF and chronic kidney disease (CKD), reducing HF‐hospitalizations and slowing the progression of renal damage in large populations, even in the absence of diabetes, with consistent efficacy across the spectrum of HF syndromes and CKD [8, 9].

In this context, and in line with recent literature [10] highlighting the need for additional cardioprotective and nephroprotective strategies, our study aimed to evaluate the association between SGLT2i exposure and cardiovascular outcomes in patients with FD, while also assessing progression to advanced kidney disease.

## 2 Methods

### 2.1 Study Design and Data Source

A retrospective observational cohort study was conducted using the TriNetX Global Collaborative Network [11], a federated real‐world research platform containing de‐identified patient‐level electronic health record data from participating healthcare organisations. The network includes healthcare organisations across multiple geographic regions, including the Americas, Europe, the Middle East, Africa and the Asia–Pacific region, thereby providing a geographically diverse study population. Diagnoses and treatments were identified using International Classification of Diseases, Tenth Revision (ICD‐10), RxNorm, VA Drug Classification and Current Procedural Terminology (CPT) codes.

### 2.2 Ethics Approval and Consent to Participate

The study was conducted using the TriNetX LIVE research network, which provides only de‐identified patient‐level data in compliance with the HIPAA Privacy Rule (45 CFR §164.514[b] [1]). Data are available exclusively as aggregated counts and statistical summaries, with no personal identifiers. A qualified expert (last updated December 2020) has certified the de‐identification process; therefore, analyses on TriNetX are not considered human subjects research and are generally exempt from Institutional Review Board (IRB) oversight. No identifiable data were accessed or re‐identified. Accordingly, this study was exempt from IRB review and informed consent requirements. All analyses complied with applicable regulations and the Declaration of Helsinki.

### 2.3 Study Population

Searches on TriNetX were conducted on 10 May 2026. Adults (≥ 18 years) with FD (ICD‐10‐CM E75.21) and estimated glomerular filtration rate (eGFR) ≥ 20 mL/min/1.73 m^2^ were eligible, thereby including patients across a broad spectrum of kidney function, including those with mild‐to‐moderate chronic kidney disease. Patients with Type 1 diabetes mellitus were excluded to minimise potential misclassification and ensure a more homogeneous study population, given possible coding inaccuracies and occasional off‐label use of SGLT2 inhibitors. In large administrative databases such as TriNetX, isolated diagnostic coding errors may persist and propagate within the dataset, further supporting this exclusion criterion.

Patients were stratified into two groups: SGLT2i‐exposed and non‐exposed patients. SGLT2i exposure was defined as at least one recorded prescription or administration of an SGLT2 inhibitor within the electronic health record. For SGLT2i‐exposed patients, the index date was defined as the first recorded SGLT2i exposure occurring at least 1 day after the first FD diagnosis. For non‐exposed patients, the index date was defined by the date of FD diagnosis in patients without any recorded SGLT2i exposure. Cohort definitions were restricted to events occurring on or after 1 January 2000.

### 2.4 Outcomes

The primary outcome was a composite of all‐cause death, acute myocardial infarction (AMI; I21–I22) and acute or acute‐on‐chronic heart failure (HF), defined as acute systolic HF (I50.21), acute‐on‐chronic systolic HF (I50.23), acute diastolic HF (I50.31), or acute‐on‐chronic diastolic HF (I50.33). Secondary outcomes included the individual components of the primary composite outcome and progression to advanced kidney disease, defined as the occurrence of chronic kidney disease Stage 4 (N18.4), Stage 5 (N18.5), end stage renal disease (N18.6), or dependence on renal dialysis (Z99.2). For each non‐fatal outcome analysis, patients with a record of the outcome under investigation before the index date were excluded to restrict the analysis to incident events and avoid capturing recurrent or prevalent cases; no corresponding exclusion was applicable to all‐cause mortality. Diagnostic codes and outcome definitions are summarised in Table S1.

### 2.5 Follow‐Up Timing

Follow‐up started 1 day after the index date. Main analyses were conducted using prespecified follow‐up windows of 2 years (730 days) and 5 years (1825 days).

### 2.6 Statistical Analysis

All statistical analyses were conducted using the TriNetX online research platform. Baseline characteristics were compared using *χ* ^2^ tests for categorical variables and independent‐samples *t*‐tests for continuous variables. To adjust for confounding, 1:1 propensity score matching (PSM) was performed using a greedy nearest‐neighbour algorithm based on multiple covariates, including age at index date, race, sex, comorbidities such as hypertensive disease, Type 2 diabetes mellitus, ischemic heart disease and HF, as well as baseline treatments including beta‐blocking agents, ACE inhibitors, angiotensin II receptor blockers, aldosterone antagonists and other potassium‐sparing agents and Fabry disease‐specific therapies (agalsidase beta, agalsidase alfa, pegunigalsidase alfa and migalastat).

Baseline covariates included in the propensity score model were assessed using all available records preceding the index date, with the assessment window ending 1 day before the index event. Renal function, assessed by eGFR, was also included in the propensity score model. Covariate balance was assessed using standardised mean differences, with values < 0.10 considered indicative of adequate balance.

Time‐to‐event outcomes were illustrated using Kaplan–Meier survival curves, and differences between groups were assessed using the log‐rank test. Hazard ratios (HRs) and 95% confidence intervals (CIs) were estimated using Cox proportional hazards models.

To assess the robustness of the association with the 5‐year primary composite outcome to potential unmeasured confounding, an *E*‐value was calculated for the post‐matching hazard ratio and for the confidence interval limit closest to the null, using the approximation for a non‐rare outcome.

To assess the robustness of the results, sensitivity analyses were performed using alternative follow‐up windows of 3 and 6 years, in addition to the primary follow‐up definition.

### 2.7 Contemporary‐Period Sensitivity Analysis

To evaluate the potential influence of secular changes in disease recognition, diagnostic practices and background therapies, we also performed a contemporary‐period sensitivity analysis restricted to patients with a recorded FD diagnosis on or after 1 January 2013, corresponding approximately to the early clinical introduction of SGLT2i for the treatment of Type 2 diabetes and their subsequent incorporation into international treatment recommendations [12, 13]. The same eligibility criteria, propensity score matching procedure, outcome definitions and 5‐year follow‐up period used in the primary analysis were applied.

### 2.8 Sensitivity Analysis Using a Stricter Fabry Disease Case Definition

To assess the robustness of the findings to potential Fabry disease phenotype misclassification, we conducted an additional sensitivity analysis using a stricter case definition requiring at least two recorded Fabry disease diagnoses separated by at least 1 month. This approach was intended to reduce the likelihood of cohort inclusion based on an isolated or potentially miscoded diagnosis. The cohort definitions, propensity score matching procedure, follow‐up duration and outcome definitions were otherwise consistent with those used in the primary analysis.

Exploratory subgroup analyses were restricted to the 5‐year primary composite outcome in the matched cohort. Subgroups included sex and age (≤ 74 vs. ≥ 75 years). Effect modification was assessed using *p* values for interaction. All tests were two‐tailed, and *p* values ≤ 0.05 were considered statistically significant. All analyses were performed within the TriNetX platform, which uses the R survival package (version 3.2).

## 3 Results

### 3.1 Baseline Characteristics

A total of 14 940 eligible patients with FD were identified, including 1028 SGLT2i‐exposed patients (mean age: 65.5 ± 12.0 years; 53.0% male) and 13 912 non‐exposed patients (mean age: 49.7 ± 22.4 years; 45.2% male). In the unmatched cohort, SGLT2i‐exposed patients were older, more often male and had a higher burden of baseline cardiorenal comorbidities (Table 1). After PSM, 732 patients remained in each group, with well‐balanced baseline characteristics (all absolute standardised mean differences < 0.1); residual imbalance was limited to infrequently recorded Fabry disease‐specific therapies (Table 1 and Figure S1).

**TABLE 1:** Baseline characteristics before and after propensity score matching among SGLT2i‐exposed and non‐exposed patients with Fabry disease.

Variable	BEFORE			AFTER		
	Fabry Sglt2i expose	Fabry Sglt2i non‐exposed	Std. Diff.	Fabry Sglt2i expose	Fabry Sglt2i non‐exposed	Std. Diff.
Age, years (mean ± SD)	65.5 ± 12.0	49.7 ± 22.4	0.883	64.2 ± 11.9	64.2 ± 12.4	0.004
White, *n* (%)	625 (60.8%)	9586 (69.0%)	0.172	455 (62.2%)	466 (63.7%)	0.031
Male, *n* (%)	545 (53.0%)	6274 (45.2%)	0.158	384 (52.5%)	366 (50.0%)	0.049
Hypertension	915 (89.0%)	3302 (23.8%)	1.747	630 (86.1%)	637 (87.0%)	0.028
Type 2 diabetes mellitus	796 (77.4%)	1312 (9.4%)	1.885	539 (73.6%)	565 (77.2%)	0.083
Ischemic heart disease	519 (50.5%)	1205 (8.7%)	1.031	294 (40.2%)	282 (38.5%)	0.034
Heart failure	310 (30.2%)	403 (2.9%)	0.789	138 (18.9%)	145 (19.8%)	0.024
Beta‐blockers	643 (62.5%)	1558 (11.2%)	1.257	389 (53.1%)	372 (50.8%)	0.047
ACE inhibitors	568 (55.3%)	1123 (8.1%)	1.177	365 (49.9%)	369 (50.4%)	0.011
Angiotensin II receptor blockers	475 (46.2%)	700 (5.0%)	1.069	259 (35.4%)	241 (32.9%)	0.052
Aldosterone antagonists	209 (20.3%)	269 (1.9%)	0.612	101 (13.8%)	89 (12.2%)	0.049
Agalsidase beta	51 (5.0%)	58 (0.4%)	0.284	20 (2.7%)	11 (1.5%)	0.086
Agalsidase alfa	19 (1.8%)	0 (0%)	0.194	10 (1.4%)	0 (0%)	0.166
Pegunigalsidase alfa	10 (1.0%)	0 (0%)	0.140	10 (1.4%)	0 (0%)	0.166
Migalastat	14 (1.4%)	15 (0.1%)	0.147	10 (1.4%)	10 (1.4%)	< 0.001
eGFR (mean ± SD)	74.0 ± 25.7	87.2 ± 22.1	0.550	77.1 ± 25.2	79.1 ± 22.7	0.082

*Note:* Standardised differences < 0.1 indicate adequate balance between groups.

Abbreviations: eGFR, estimated glomerular filtration rate; PSM, propensity score matching; SD, standard deviation.

### 3.2 Survival Analysis

At 2 years after PSM, SGLT2i‐exposed patients showed a lower risk of the composite outcome (HR 0.61, 95% CI 0.38–0.97, Figure 1A) and all‐cause death (HR 0.56, 95% CI 0.33–0.95) compared with non‐exposed patients. No significant differences were observed for acute/acute‐on‐chronic heart failure or progression to advanced kidney disease (Table 2 and Figure 1A). The analysis of acute myocardial infarction after PSM was not displayed because the event count was too low.

**FIGURE 1:**
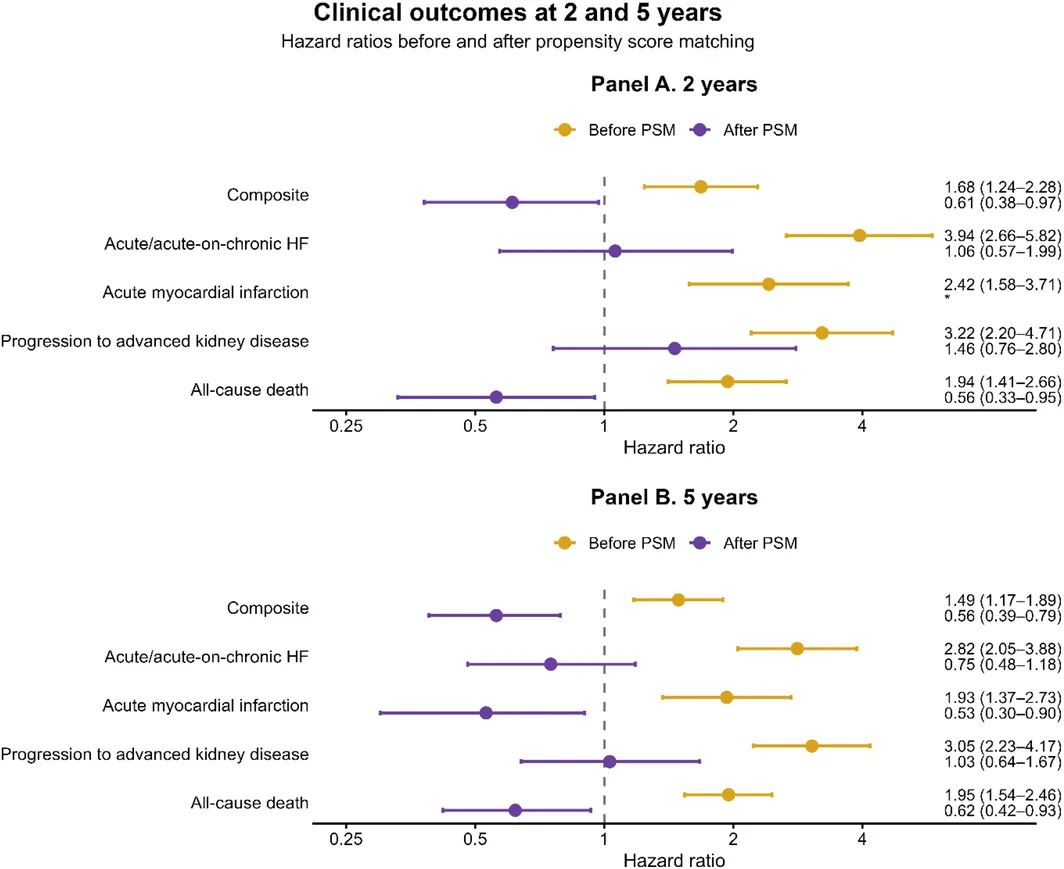
Forest plot showing hazard ratios (HRs) with 95% confidence intervals (CIs) for SGLT2i‐exposed versus non‐exposed patients before and after PSM. (A) 2‐year follow‐up. (B) 5‐year follow‐up. Clinical outcomes at 2 and 5 years before and after propensity score matching. (A) Shows outcomes at 2 years and (B) at 5 years. Hazard ratios (HRs) with 95% confidence intervals are reported for each outcome before and after propensity score matching. The dashed vertical line indicates HR = 1. HF indicates heart failure. *Myocardial infarction results after propensity score matching at 2 years were not displayed because the event count was too low.

**TABLE 2:** Two‐year clinical outcomes in propensity score–matched patients with Fabry disease according to SGLT2i exposure.

Outcome	BEFORE PSM			AFTER PSM		
	Number of events	Risk (%)	HR (95% CI)	Number of events	Risk (%)	HR (95% CI)
Composite	45/798 versus 497/13 514	5.6% versus 3.7%	1.68 (1.24–2.28)	26/619 versus 49/642	4.2% versus 7.6%	0.61 (0.38–0.97)
Acute/acute‐on‐chronic heart failure	31/887 versus 133/13 796	3.5% versus 1.0%	3.94 (2.66–5.82)	19/672 versus 20/690	2.8% versus 2.9%	1.06 (0.57–1.99)
Acute myocardial infarction	24/875 versus 172/13 626	2.7% versus 1.3%	2.42 (1.58–3.71)	^a^	^a^	^a^
Progression to advanced kidney disease	32/949 versus 155/13 754	3.4% versus 1.1%	3.22 (2.20–4.71)	21/693 versus 16/711	3.0% versus 2.3%	1.46 (0.76–2.80)
All‐cause death	43/1028 versus 329/13 912	4.2% versus 2.4%	1.94 (1.41–2.66)	21/732 versus 41/732	2.9% versus 5.6%	0.56 (0.33–0.95)

*Note:* A statistical method used to balance baseline characteristics between groups by matching patients with a similar probability of exposure. Before matching, 1028 SGLT2i‐exposed patients and 13 912 non‐exposed patients were included; after matching, 732 patients remained in each group.

Abbreviation: PSM, propensity score matching.

^a^ Myocardial infarction results after matching were not displayed because the event count was too low. Patient numbers may vary across outcomes (composite outcome, acute/acute‐on‐chronic heart failure, acute myocardial infarction, and progression to advanced kidney disease) due to the exclusion of individuals with the specific outcome prior to the index date/time window.

At 5 years after PSM, SGLT2i‐exposed patients showed a lower risk of the composite outcome (HR 0.56, 95% CI 0.39–0.79, Figure 1B), all‐cause death (HR 0.62, 95% CI 0.42–0.93) and acute myocardial infarction (HR 0.53, 95% CI 0.30–0.90) compared with non‐exposed patients (Table 3). No significant differences were observed for acute/acute‐on‐chronic heart failure or progression to advanced kidney disease (Table 3 and Figure 1B). As shown in Figure 2, Kaplan–Meier curves for the composite outcome diverged between SGLT2i‐exposed and non‐exposed patients at 5 years. For the 5‐year primary composite outcome, the *E*‐value was 2.37 for the post‐matching point estimate and 1.63 for the confidence interval limit closest to the null.

**TABLE 3:** Five‐year clinical outcomes in propensity score–matched patients with Fabry disease according to SGLT2i exposure (exposed vs. non‐exposed).

Outcome	BEFORE PSM			AFTER PSM		
	Number of events	Risk (%)	HR (95% CI)	Number of events	Risk (%)	HR (95% CI)
Composite	72/798 versus 1119/13 514	9.0% versus 8.3%	1.49 (1.17–1.89)	43/619 versus 105/642	6.9% versus 16.4%	0.56 (0.39–0.79)
Acute/acute‐on‐chronic heart failure	43/887 versus 330/13 796	4.8% versus 2.4%	2.82 (2.05–3.88)	29/672 versus 53/690	4.3% versus 7.7%	0.75 (0.48–1.18)
Acute myocardial infarction	35/875 versus. 399/13 626	4.0% versus 2.9%	1.93 (1.37–2.73)	18/653 versus 48/669	2.8% versus 7.2%	0.53 (0.30–0.90)
Progression to advanced kidney disease	46/949 versus 287/13 754	4.8% versus 2.1%	3.05 (2.23–4.17)	30/693 versus 39/711	4.3% versus 5.5%	1.03 (0.64–1.67)
All‐cause death	78/1028 versus 770/13 912	7.6% versus 5.5%	1.95 (1.54–2.46)	36/732 versus 74/732	4.9% versus 10.1%	0.62 (0.42–0.93)

*Note:* Events are reported as the number of events among SGLT2i‐exposed versus non‐exposed patients. Risk is expressed as the percentage of SGLT2i‐exposed versus non‐exposed patients experiencing the outcome. For the 5‐year analysis, 1028 SGLT2i‐exposed patients and 13 912 non‐exposed patients were included before matching; after matching, 732 patients remained in each group. Patient numbers may vary across outcomes (composite outcome, acute/acute‐on‐chronic heart failure, acute myocardial infarction and progression to advanced kidney disease) due to the exclusion of individuals with the specific outcome prior to the index date/time window.

Abbreviation: PSM, propensity score matching.

**FIGURE 2:**
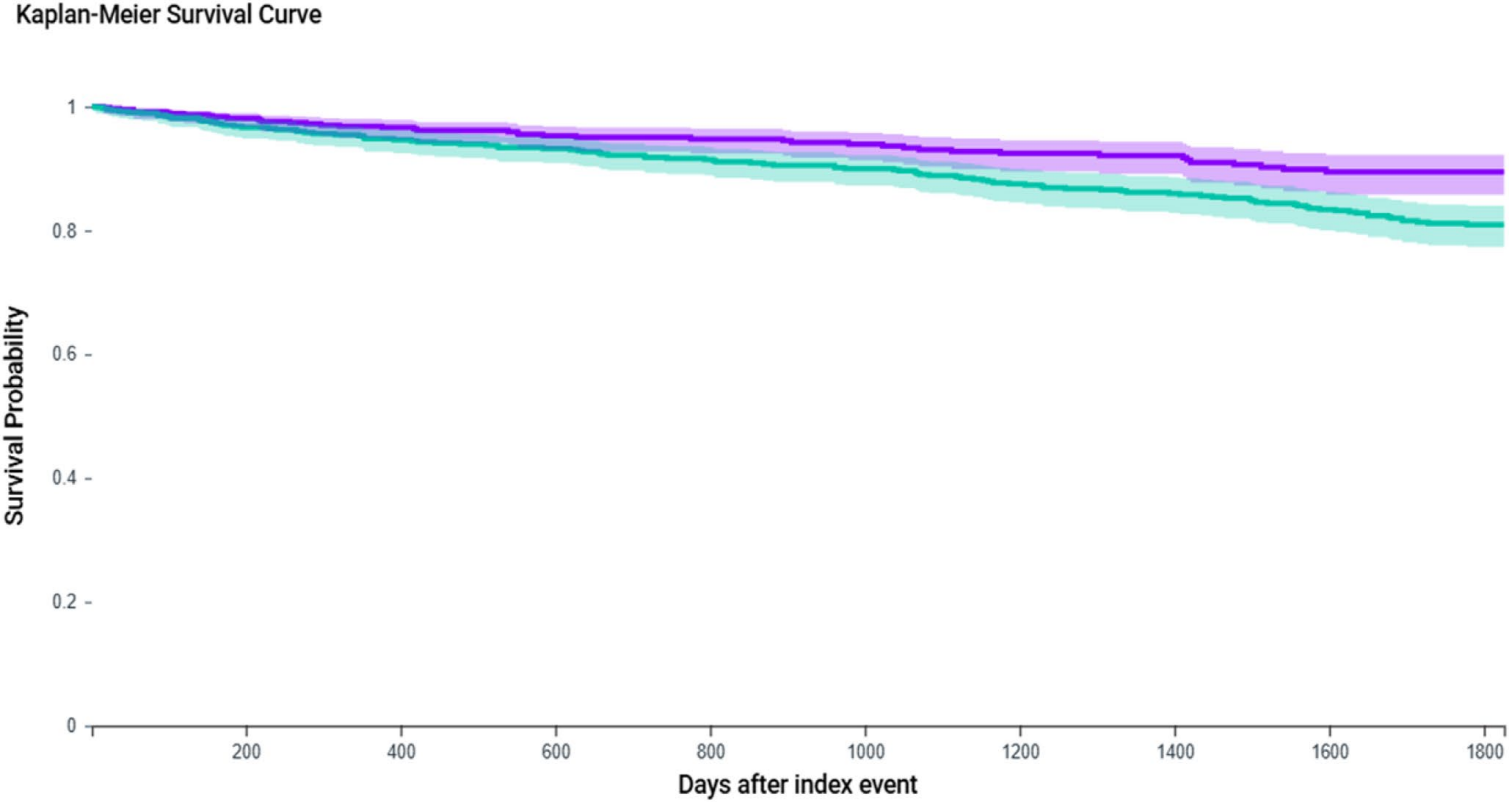
Five‐year post–propensity score‐matched Kaplan–Meier curves for the composite outcome between SGLT2i‐exposed and non‐exposed patients. The purple line represents SGLT2i‐exposed patients with Fabry disease, whereas the green line represents non‐exposed patients with Fabry disease.

### 3.3 Sensitivity Analyses at 3 and 6 Years

Sensitivity analyses using alternative follow‐up durations of 3 and 6 years yielded results broadly consistent with the main analysis, showing a lower risk of the composite outcome among SGLT2i‐exposed patients (3 years: HR 0.61, 95% CI 0.40–0.93; 6 years: HR 0.65, 95% CI 0.47–0.90). A similar pattern was observed for all‐cause death, which remained significantly lower in SGLT2i users at both time points (3 years: HR 0.61, 95% CI 0.39–0.96; 6 years: HR 0.63, 95% CI 0.43–0.93) (Table S2 and Figure S2).

### 3.4 Contemporary‐Period Sensitivity Analysis

The contemporary‐period sensitivity analysis included 564 SGLT2i‐exposed and 9103 non‐exposed patients before matching, with 441 patients retained in each cohort after PSM. Matching substantially improved covariate balance, although residual imbalance remained for eGFR and the infrequently recorded use of pegunigalsidase alfa (Table S4). At 5 years, SGLT2i exposure remained associated with a lower risk of the primary composite outcome compared with non‐exposure (HR 0.60, 95% CI 0.39–0.94), consistent with the primary analysis (Figure S3).

### 3.5 Sensitivity Analysis Using a Stricter Fabry Disease Case Definition

In the sensitivity analysis using a stricter Fabry disease case definition, 550 SGLT2i‐exposed and 6104 non‐exposed patients were included before matching, with 299 patients retained in each cohort after PSM. SGLT2i exposure remained associated with a lower risk of the primary composite outcome (HR 0.51, 95% CI 0.28–0.94), consistent with the primary analysis. No significant association was observed for progression to advanced kidney disease (HR 1.72, 95% CI 0.90–3.29) (Tables S5 and S6).

### 3.6 Subgroup Analyses

Exploratory subgroup analyses for the 5‐year composite outcome in the matched cohort showed a consistent association between SGLT2i exposure and lower event risk across sex and age groups. Specifically, hazard ratios were below 1 in both males and females, as well as in patients aged ≤ 74 and ≥ 75 years. No significant effect modification was observed by sex (*p* for interaction = 0.14) or age (*p* for interaction = 0.92) (Figure 3 and Table S3).

**FIGURE 3:**
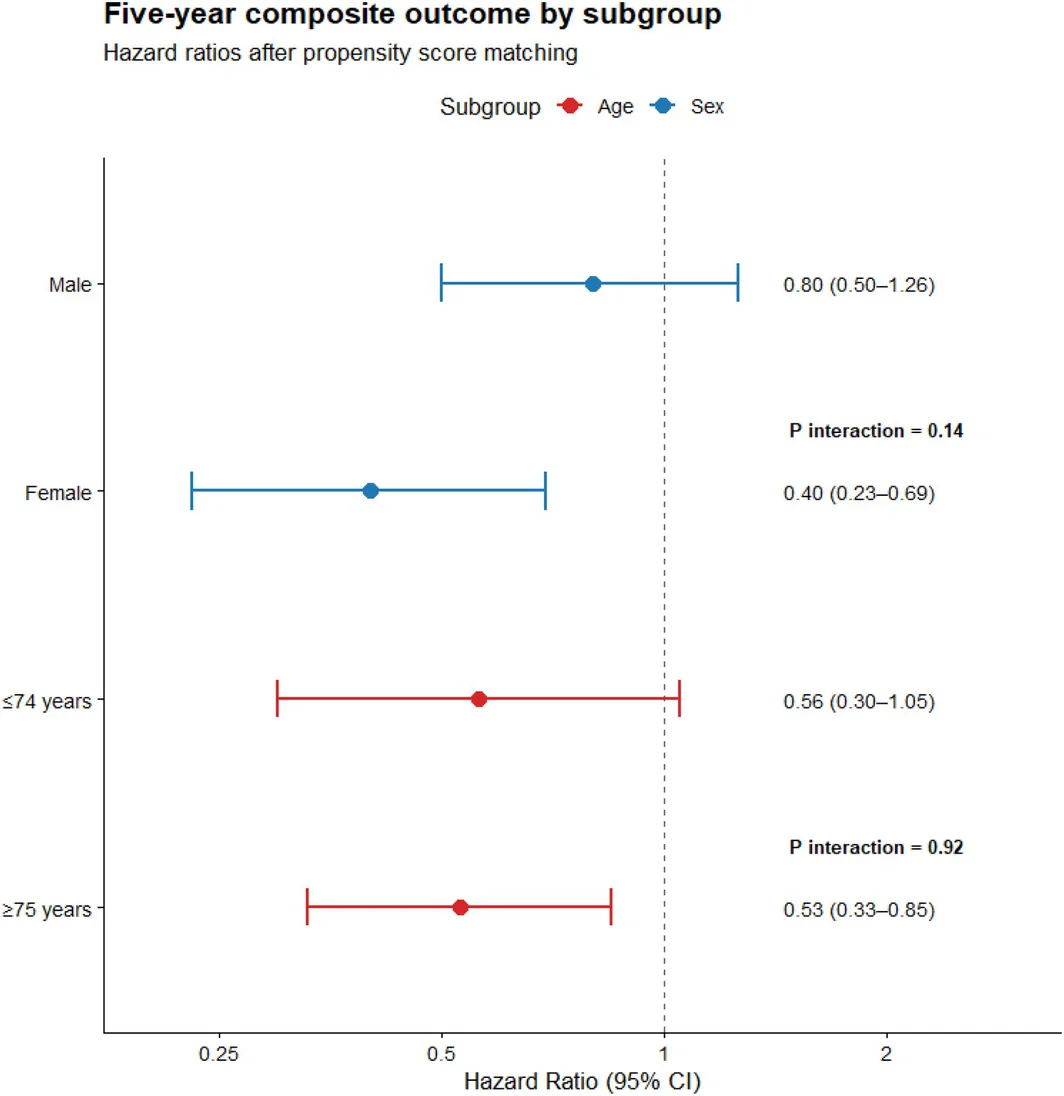
Forest plot of subgroup analyses for the 5‐year composite outcome before and after propensity score matching. HRs with 95% CIs for the 5‐year composite outcome are shown across predefined subgroups before and after PSM. *p* values for interaction refer to subgroup categories in the matched analysis: Male versus female and age ≤ 74 versus ≥ 75 years.

## 4 Discussion

The main findings of the present study can be summarised as follows: (i) Among patients with FD, SGLT2i‐exposed patients had a lower risk of the composite outcome and all‐cause death, as well as myocardial infarction at 5 years compared with non‐exposed patients. (ii) These associations were already evident at 2 years and remained overall consistent across sensitivity analyses using alternative follow‐up windows of 3 and 6 years, supporting the robustness of the main findings over time. (iii) Exploratory subgroup analyses showed that the association between SGLT2i exposure and a lower risk of the composite endpoint at 5 years was broadly consistent across sex and age categories, with no significant evidence of interaction.

Our findings appear clinically relevant because cardiac and renal involvement are among the major determinants of morbidity and long‐term prognosis in FD. Before PSM, SGLT2i‐exposed patients appeared clinically more vulnerable than non‐exposed patients, suggesting a non‐random treatment allocation in routine practice. This pattern is consistent with treatment channelling towards patients with a higher cardiorenal burden and should be considered when interpreting crude baseline differences between groups.

Progression towards heart failure, ischemic events, deterioration of renal function and death represents a central component of the natural history of FD, even in patients receiving disease‐specific therapies [14]. Accordingly, the composite endpoint used in the present study was selected to capture major clinically relevant cardiovascular events associated with adverse prognosis in FD and reliably identifiable within electronic health records. In this context, the association between SGLT2i exposure and lower risks of the composite outcome and all‐cause mortality, with an additional association with lower AMI risk at 5 years, may be clinically relevant. Overall, these findings support further investigation of SGLT2i exposure in patients with FD, particularly in prospective studies and dedicated Fabry disease cohorts.

Importantly, the association with the primary composite outcome remained consistent in the contemporary‐period sensitivity analysis restricted to patients with a recorded FD diagnosis from 2013 onwards. Although the smaller sample size reduced the precision of estimates for individual outcomes, the magnitude and direction of the primary association were similar to those observed in the main analysis. This finding reduces concern that the observed association was entirely attributable to secular changes in disease recognition and background treatment; nevertheless, residual calendar‐time confounding and other time‐related biases cannot be excluded.

Similarly, the association remained consistent when a stricter Fabry disease case definition requiring at least two diagnoses separated by at least 1 month was applied, supporting the robustness of the findings and reducing concerns regarding potential diagnostic misclassification. However, estimates for individual outcomes were limited by the small number of events.

A further methodological consideration relates to the different definition of the index date between the cohorts. Exposed patients were indexed at the first recorded SGLT2 inhibitor exposure after FD diagnosis, whereas non‐exposed patients were indexed at FD diagnosis. Consequently, immortal time bias and differential time‐at‐risk cannot be excluded, and PSM could not correct this temporal misalignment. Therefore, the observed associations should be interpreted cautiously and not as evidence of a causal treatment effect.

The observed associations are also biologically plausible. SGLT2i reduce congestion and filling pressures, improve hemodynamic status and cardiac energy metabolism, attenuate inflammation and oxidative stress and may promote more favourable myocardial remodelling [15, 16]. In addition, recent evidence suggests that SGLT2i may exert favourable effects on cardiac structure, including reductions in left ventricular mass and epicardial adipose tissue, although these observations have been reported primarily in patients with heart failure and Type 2 diabetes [17]. Considering that hypertrophy, diastolic dysfunction, and fibrosis are key drivers of progression towards heart failure and arrhythmias in FD, these mechanisms provide biological plausibility for the observed associations beyond disease‐specific therapies [1].

An important consideration is the high prevalence of Type 2 diabetes among SGLT2 inhibitor‐exposed patients, as diabetes likely represented a major indication for treatment. Although diabetes was included in the propensity score matching procedure, residual confounding related to diabetes severity, treatment indication and longitudinal glycaemic control cannot be excluded. Consequently, the observed associations may partly reflect the established cardiometabolic benefits of SGLT2 inhibitors in patients with diabetes rather than a Fabry‐specific myocardial effect. Further studies are needed to determine whether these associations extend to patients with Fabry disease without diabetes.

Concerning the second point, the pattern of results across different time horizons is itself informative. At 2 years, the association was already evident for the composite outcome and for all‐cause death, whereas at 5 years it appeared stronger and extended to AMI. Sensitivity analyses using alternative follow‐up windows of 3 and 6 years confirmed this pattern, suggesting that the main findings are not driven solely by the choice of the 2‐ and 5‐year time points. Overall, these data are consistent with an association that appears more evident over time.

This temporal pattern is in line with evidence from major cardiovascular outcome trials of SGLT2i. In EMPA‐REG OUTCOME, empagliflozin reduced MACE and cardiovascular mortality over a median follow‐up of approximately 3.1 years [18]; in the CANVAS Program, canagliflozin reduced MACE over a mean follow‐up of approximately 3.6 years [19]; and in DECLARE–TIMI 58, with a median follow‐up of 4.2 years, dapagliflozin reduced the composite of cardiovascular death or hospitalisation for heart failure, without a significant reduction in MACE [20]. Taken together, and further supported by subsequent class meta‐analyses, these data suggest that the cardiovascular effects of SGLT2i have been shown to emerge and consolidate over the medium to long term, with greater consistency for heart failure and cardiorenal outcomes than for purely ischemic endpoints. The second finding therefore supports the temporal robustness of the observed association and its consistency with the efficacy profile previously described for the SGLT2i class. However, this finding should be interpreted with caution, *as the study was not powered for this specific endpoint and residual confounding cannot be excluded*.

Exploratory subgroup analyses showed that the association between SGLT2i exposure and a lower risk of the composite endpoint at 5 years was broadly consistent across sex and age strata, with no significant evidence of statistical interaction. This finding appears particularly relevant in the context of Fabry cardiomyopathy, where age and sex are major determinants of myocardial phenotype and progression of cardiac involvement, with earlier onset of structural and tissue abnormalities typically observed in males [21]. In this setting, the absence of apparent effect heterogeneity across these key demographic strata may support the potential relevance of the observed association across different patient profiles. This pattern is also consistent with the broader SGLT2i literature, in which cardiovascular benefits have generally been similar across sex‐ and age‐defined subgroups, without clear evidence of clinically meaningful interaction, as also reported in the meta‐analysis by Zannad et al. [22]. Although these analyses should be interpreted cautiously given their exploratory nature, the third finding suggests that the observed signal is not limited to a specific demographic subgroup but remains broadly consistent across the main clinically relevant strata.

SGLT2i users also had a lower risk of AMI at 5 years. This finding should be interpreted with caution, as reduction in AMI has been less consistently demonstrated than benefits on HF and cardiorenal outcomes. However, previous evidence supports a possible anti‐ischemic effect of the class, with meta‐analytic data from cardiovascular outcome trials suggesting a lower risk of AMI and a subsequent analysis from EMPA‐REG OUTCOME reporting fewer total AMI events with empagliflozin [23, 24]. Additional, albeit less direct, support comes from observational evidence in selected high‐risk ischemic populations, including patients with Type 2 diabetes and ischemic heart disease undergoing CABG, in whom chronic SGLT2i therapy was associated with more favourable 5‐year clinical outcomes [25]. Taken together, these data suggest that the lower risk of myocardial infarction observed in our study is plausible, although it should be interpreted cautiously.

The marked differences between the unadjusted and post‐PSM analyses are also important for the interpretation of the primary finding. The pre‐matching results may, at least in part, reflect the clinical complexity of FD. Given the marked phenotypic heterogeneity of FD [26] and the adverse prognostic impact of multiorgan involvement, particularly combined cardiac and renal disease [27], patients treated with SGLT2i in the unmatched cohort may represent a subgroup with greater baseline severity. This may account for the higher event rates observed before matching. After PSM, once baseline characteristics were more adequately balanced, the association became more interpretable, suggesting that the unadjusted findings were likely influenced by confounding by indication and underlying disease severity. However, because matching retained only a selected subset of the original non‐exposed population with sufficient covariate overlap, the post‐matching findings primarily apply to the matched cohort and may not be generalizable to the broader population of patients with a recorded diagnosis of FD.

Overall, these findings support the hypothesis that SGLT2i exposure may be associated with more favourable cardiovascular outcomes in patients with FD, including lower risks of mortality and myocardial infarction. By contrast, no significant association with progression to advanced kidney disease was observed at either 2 or 5 years. Therefore, the present findings do not provide evidence of a renal benefit associated with SGLT2i exposure. Nevertheless, the renal endpoint used in this study captured advanced kidney disease events and may not reflect earlier or more gradual progression of Fabry nephropathy. The potential renal association remains to be determined in studies directly assessing longitudinal eGFR trajectories and albuminuria. Prospective studies or dedicated registries with detailed clinical and laboratory data will therefore be necessary to confirm these associations, determine whether they reflect causal treatment effects, evaluate their consistency across sex, age and cardiac or renal phenotypes and investigate the potential role and timing of SGLT2i alongside disease‐specific therapies.

### 4.1 Limitations

This study has several limitations. First, its retrospective observational design precludes causal inference and does not eliminate the possibility of residual confounding, despite the use of propensity score matching. No negative control outcome was prespecified, and the retrospective selection of a post hoc negative control without sufficient clinical and methodological justification was considered potentially misleading. Consequently, residual unmeasured confounding cannot be excluded. Second, exposure, covariates and outcomes were ascertained from routinely collected coded electronic health‐record data and are therefore subject to potential misclassification. Fabry disease was identified using diagnostic coding within the TriNetX network, and confirmation through genotype, α‐galactosidase A activity, lyso‐Gb3 levels, or other disease‐specific biomarkers was not consistently available. Therefore, diagnostic misclassification of Fabry disease cannot be completely excluded, which is particularly relevant given the rarity and clinical heterogeneity of the condition. In addition, the available dataset did not provide granular information on treatment adherence, dose, treatment duration, prescribing indication, longitudinal glycaemic control, or cause‐specific mortality. Formal comparative competing‐risk analyses using Fine–Grey subdistribution hazard models could not be implemented within the TriNetX analytical framework; therefore, the potential competing effect of mortality on non‐fatal outcomes could not be fully evaluated. Furthermore, because exposure was defined according to the first recorded SGLT2 inhibitor prescription after Fabry disease diagnosis, some degree of immortal time bias cannot be completely excluded despite the analytical approach adopted. Another important limitation is the incomplete availability of several disease‐specific variables that are highly relevant in FD, including genotype, phenotypic subtype and severity, extent of myocardial fibrosis, albuminuria, timing and type of disease‐specific therapy and disease biomarkers [28]. This is particularly relevant in FD, where clinical expression is markedly heterogeneous and both monitoring strategies and treatment decisions are influenced by phenotype, organ involvement and timing of therapeutic intervention [29]. Finally, because the majority of SGLT2 inhibitor‐exposed patients had Type 2 diabetes, residual confounding related to diabetes and treatment indication cannot be completely excluded. In addition, the rarity of Fabry disease limited the feasibility and statistical power of some exploratory analyses, particularly those according to diabetes status. Overall, these considerations warrant cautious interpretation of the findings and limit both their generalizability and causal inference.

## 5 Conclusion

In this real‐world cohort of patients with FD, exposure to SGLT2 inhibitors was associated with a lower risk of the composite endpoint and all‐cause mortality, with additional favourable signals for myocardial infarction over longer follow‐up. These findings should be considered hypothesis‐generating and warrant confirmation in prospective studies and dedicated Fabry disease cohorts or registries.

## Author Contributions

**Leandra Serio:** writing – original draft, writing – review and editing, visualisation, conceptualisation. **Michele Rossi**, **Amir Askarinejad**, **Enrico Tartaglia**, **Tommaso Bucci**, **Enrica Marrone**, **Claudio Ferri**, **Dinesh K. Kalra:** writing review and editing, methodology. **Gregory Y. H. Lip:** conceptualization, project administration, supervision, writing – review and editing.

## Funding

The authors have nothing to report.

## Conflicts of Interest

G.Y.H.L. reports institutional consultancy and/or speaker roles for BMS/Pfizer, Boehringer Ingelheim, Anthos, Huawei (No fees are received personally). All the disclosures happened outside the submitted work. He is a National Institute for Health and Care Research (NIHR) Senior Investigator and co‐PI of the AFFIRMO project on multimorbidity in AF (grant agreement no. 899871), TARGET project on digital twins for personalised management of atrial fibrillation and stroke (grant agreement no. 101136244) and ARISTOTELES project on artificial intelligence for management of chronic long‐term conditions (grant agreement no. 101080189), which are all funded by the EU's Horizon Europe Research and Innovation program. The other authors declare no conflicts of interest.

## Supporting information


**Table S1:** Codes for cohort definitions, exclusions, exposure and outcomes.
**Table S2:** Comparison of hazard ratios (95% CIs) for clinical outcomes at 3 and 6 years before and after propensity score matching.
**Table S3:** Subgroup analyses of the 5‐year composite outcome: hazard ratios before and after propensity score matching, and interaction *p*‐values after matching.
**Table S4:** Baseline characteristics before and after propensity score matching in the contemporary‐period sensitivity analysis restricted to Fabry disease diagnoses recorded from 2013 onwards.
**Table S5:** Baseline characteristics in the sensitivity analysis requiring ≥ 2 Fabry disease diagnostic records.
**Table S6:** Outcomes in sensitivity analysis.
**Figure S1:** Love plot showing covariate balance before and after propensity score matching.
**Figure S2:** Forest plot of outcomes at 3 and 6 years before and after propensity score matching (PSM).
**Figure S3:** Five‐year clinical outcomes in the contemporary‐period sensitivity analysis.

## Data Availability

The data that support the findings of this study were obtained from the TriNetX Global Collaborative Network. The underlying individual‐level patient data are not publicly available and cannot be shared by the authors because they are maintained by the participating healthcare organisations and accessed through the TriNetX platform under applicable data‐use agreements. Aggregated results supporting the findings of this study are available from the corresponding author upon reasonable request.

## References

[1] O. Azevedo , F. Cordeiro , M. F. Gago , et al., “Fabry Disease and the Heart: A Comprehensive Review,” International Journal of Molecular Sciences 22 (2021): 22, 10.3390/ijms22094434.

[2] M. Siegenthaler , U. Huynh‐Do , P. Krayenbuehl , et al., “Impact of Cardio‐Renal Syndrome on Adverse Outcomes in Patients With Fabry Disease in a Long‐Term Follow‐Up,” International Journal of Cardiology 249 (2017): 261–267, 10.1016/j.ijcard.2017.09.027. 28964554

[3] N. Madan and D. Kalra , “Clinical Evaluation of Infiltrative Cardiomyopathies Resulting in Heart Failure With Preserved Ejection Fraction,” Reviews in Cardiovascular Medicine 21 (2020): 181–190, 10.31083/j.rcm.2020.02.65. 32706207

[4] M. Umer , M. Motwani , J. L. Jefferies , S. F. Nagueh , and D. K. Kalra , “Cardiac Involvement in Fabry Disease and the Role of Multimodality Imaging in Diagnosis and Disease Monitoring,” Current Problems in Cardiology 48 (2023): 101439, 10.1016/j.cpcardiol.2022.101439. 36202174

[5] I. Simonetta , A. Tuttolomondo , T. Di Chiara , et al., “Genetics and Gene Therapy of Anderson‐Fabry Disease,” Current Gene Therapy 18 (2018): 96–106, 10.2174/1566523218666180404161315. 29618309

[6] M. Umer and D. K. Kalra , “Cardiac MRI in Fabry Disease,” Frontiers in Cardiovascular Medicine 9 (2022): 1075639, 10.3389/fcvm.2022.1075639. PMC9931723 36818911

[7] M. Umer and D. K. Kalra , “Treatment of Fabry Disease: Established and Emerging Therapies,” Pharmaceuticals 16 (2023): 320, 10.3390/ph16020320. PMC9967779 37259462

[8] M. S. Usman , D. L. Bhatt , I. Hameed , et al., “Effect of SGLT2 Inhibitors on Heart Failure Outcomes and Cardiovascular Death Across the Cardiometabolic Disease Spectrum: A Systematic Review and Meta‐Analysis,” Lancet Diabetes and Endocrinology 12 (2024): 447–461, 10.1016/s2213-8587(24)00102-5. 38768620

[9] Y. Battaglia , F. Bulighin , L. Zerbinati , N. Vitturi , G. Marchi , and G. Carraro , “Dapaglifozin on Albuminuria in Chronic Kidney Disease Patients With FabrY Disease: The DEFY Study Design and Protocol,” Journal of Clinical Medicine 12 (2023): 12, 10.3390/jcm12113689. PMC10253838 37297884

[10] H. Okamoto , S. Goto , M. Fujita , and H. Fujii , “Renoprotective Effects of SGLT2 Inhibitors in Patients With Fabry Disease,” Molecular Genetics and Metabolism Reports 45 (2025): 101271, 10.1016/j.ymgmr.2025.101271. PMC12554905 41158984

[11] TriNetX L , “TriNetX Global Healthcare Network,” 2026, trinetx.com.

[12] W. T. Cefalu , L. A. Leiter , K. H. Yoon , et al., “Efficacy and Safety of Canagliflozin Versus Glimepiride in Patients With Type 2 Diabetes Inadequately Controlled With Metformin (CANTATA‐SU): 52 Week Results From a Randomised, Double‐Blind, Phase 3 Non‐Inferiority Trial,” Lancet 382 (2013): 941–950, 10.1016/s0140-6736(13)60683-2. 23850055

[13] S. E. Inzucchi , R. M. Bergenstal , J. B. Buse , et al., “Management of Hyperglycemia in Type 2 Diabetes, 2015: A Patient‐Centered Approach: Update to a Position Statement of the American Diabetes Association and the European Association for the Study of Diabetes,” Diabetes Care 38 (2015): 140–149, 10.2337/dc14-2441. 25538310

[14] M. Pieroni , C. Zocchi , and M. Ciabatti , “Cardiac Involvement in Fabry Disease: Recent Advances, Unresolved Issues, and Unmet Needs,” European Heart Journal Supplements: Journal of the European Society of Cardiology 27 (2025): i51–i55, 10.1093/eurheartjsupp/suae104. PMC11836711 39980776

[15] M. R. Cowie and M. Fisher , “SGLT2 Inhibitors: Mechanisms of Cardiovascular Benefit Beyond Glycaemic Control,” Nature Reviews Cardiology 17 (2020): 761–772, 10.1038/s41569-020-0406-8. 32665641

[16] Y. Zhang and Q. Han , “A Review of Cardiovascular Benefits of SGLT2 Inhibitors,” Medicine 101 (2022): e30310, 10.1097/md.0000000000030310. PMC10980435 36086785

[17] M. Alghamdi , A. Dihoum , K. Hakami , et al., “Dapagliflozin Reduces Epicardial Adipose Tissue in Patients With Heart Failure and Type 2 Diabetes,” Diabetes, Obesity and Metabolism 27 (2025): 7561–7569, 10.1111/dom.70164. PMC12587262 41053997

[18] S. Verma , L. A. Leiter , B. Zinman , et al., “Time to Cardiovascular Benefits of Empagliflozin: A Post Hoc Observation From the EMPA‐REG OUTCOME Trial,” ESC Heart Failure 8 (2021): 2603–2607, 10.1002/ehf2.13374. PMC8318423 34132492

[19] B. Neal , V. Perkovic , K. W. Mahaffey , et al., “Canagliflozin and Cardiovascular and Renal Events in Type 2 Diabetes,” New England Journal of Medicine 377 (2017): 644–657, 10.1056/NEJMoa1611925. 28605608

[20] S. D. Wiviott , I. Raz , M. P. Bonaca , et al., “Dapagliflozin and Cardiovascular Outcomes in Type 2 Diabetes,” New England Journal of Medicine 380 (2019): 347–357, 10.1056/NEJMoa1812389. 30415602

[21] S. Nordin , R. Kozor , K. Medina‐Menacho , et al., “Proposed Stages of Myocardial Phenotype Development in Fabry Disease,” JACC: Cardiovascular Imaging 12 (2019): 1673–1683, 10.1016/j.jcmg.2018.03.020. 29778854

[22] F. Zannad , J. P. Ferreira , S. J. Pocock , et al., “SGLT2 Inhibitors in Patients With Heart Failure With Reduced Ejection Fraction: A Meta‐Analysis of the EMPEROR‐Reduced and DAPA‐HF Trials,” Lancet 396 (2020): 819–829, 10.1016/s0140-6736(20)31824-9. 32877652

[23] T. A. Zelniker , S. D. Wiviott , I. Raz , et al., “SGLT2 Inhibitors for Primary and Secondary Prevention of Cardiovascular and Renal Outcomes in Type 2 Diabetes: A Systematic Review and Meta‐Analysis of Cardiovascular Outcome Trials,” Lancet 393 (2019): 31–39, 10.1016/s0140-6736(18)32590-x. 30424892

[24] D. Fitchett , B. Zinman , S. E. Inzucchi , et al., “Effect of Empagliflozin on Total Myocardial Infarction Events by Type and Additional Coronary Outcomes: Insights From the Randomized EMPA‐REG OUTCOME Trial,” Cardiovascular Diabetology 23 (2024): 248, 10.1186/s12933-024-02328-6. PMC11241944 38992713

[25] C. Sardu , M. Massetti , N. Testa , et al., “Effects of Sodium‐Glucose Transporter 2 Inhibitors (SGLT2‐I) in Patients With Ischemic Heart Disease (IHD) Treated by Coronary Artery Bypass Grafting via MiECC: Inflammatory Burden, and Clinical Outcomes at 5 Years of Follow‐Up,” Frontiers in Pharmacology 12 (2021): 777083, 10.3389/fphar.2021.777083. PMC8634684 34867407

[26] A. Jovanovic , E. Miller‐Hodges , F. Castriota , et al., “Clinical Efficacy and Real‐World Effectiveness of Fabry Disease Treatments: A Systematic Literature Review,” Journal of Clinical Medicine 14 (2025): 14, 10.3390/jcm14145131. PMC12295217 40725823

[27] X. Yin , Z. Song , X. Sun , et al., “Arrhythmias and Clinical Outcomes in Fabry Disease With Cardiac and Renal Involvement,” Orphanet Journal of Rare Diseases 20 (2025): 561, 10.1186/s13023-025-04079-3. PMC12587517 41194135

[28] A. Burlina , E. Brand , D. Hughes , et al., “An Expert Consensus on the Recommendations for the Use of Biomarkers in Fabry Disease,” Molecular Genetics and Metabolism 139 (2023): 107585, 10.1016/j.ymgme.2023.107585. 37207471

[29] M. Biegstraaten , R. Arngrímsson , F. Barbey , et al., “Recommendations for Initiation and Cessation of Enzyme Replacement Therapy in Patients With Fabry Disease: The European Fabry Working Group Consensus Document,” Orphanet Journal of Rare Diseases 10 (2015): 36, 10.1186/s13023-015-0253-6. PMC4383065 25885911

