# Supplementary material for: SGLT2 Inhibitors and Cardiovascular Outcomes in Patients With Fabry Disease: Observations From a Federated Research Network
Source: Diabetes Obes Metab. 2026 Sep 8;28(12):11206–16. doi: 10.1111/dom.71324 (PMC13731061; doi:10.1111/dom.71324)
Supplement: Supplementary file 1 [file DOM-28-11206-s001.docx]

**SGLT2 Inhibitors and Cardiovascular Outcomes in Patients with Fabry Disease: Observations from a Federated Research Network**

Leandra Serio , Michele Rossi , Amir Askarinejad, Enrico Tartaglia, Tommaso Bucci , Enrica Marrone, Dinesh K. Kalra, Claudio Ferri, Gregory Y.H. Lip

**Supplementary material**

**Supplementary Table 1.** Codes for cohort definitions, exclusions, exposure, and outcomes

| Section | Outcome category | Outcome name | Coding system | Code | Description | Use in analysis |
| --- | --- | --- | --- | --- | --- | --- |
| Cohort definition | Inclusion | Fabry disease | ICD-10-CM | E75.21 | Fabry (-Anderson) disease | Inclusion |
| Cohort definition | Exposure | SGLT2i | ATC | A10BK | Sodium-glucose cotransporter-2 inhibitors | Exposed cohort definition |
| Cohort definition | Exclusion | Type 1 diabetes mellitus | ICD-10-CM | E10 | Type 1 diabetes mellitus | Exclusion |
| Cohort definition | Exclusion | eGFR <20 mL/min/1.73 m² | TNX | 8001 | MDRD-based eGFR | Exclusion |
| Outcome definition | Primary outcome | Composite | ICD-10-CM | I21 | Acute myocardial infarction | Composite endpoint |
| Outcome definition | Primary outcome | Composite | ICD-10-CM | I22 | Subsequent STEMI/NSTEMI myocardial infarction | Composite endpoint |
| Outcome definition | Primary outcome | Composite | Demographics | — | Deceased | Composite endpoint |
| Outcome definition | Primary outcome | Composite | ICD-10-CM | I50.21 | Acute systolic heart failure | Composite endpoint |
| Outcome definition | Primary outcome | Composite | ICD-10-CM | I50.23 | Acute on chronic systolic heart failure | Composite endpoint |
| Outcome definition | Primary outcome | Composite | ICD-10-CM | I50.31 | Acute diastolic heart failure | Composite endpoint |
| Outcome definition | Primary outcome | Composite | ICD-10-CM | I50.33 | Acute on chronic diastolic heart failure | Composite endpoint |
| Outcome definition | Secondary outcome | Acute/acute-on-chronic HF | ICD-10-CM | I50.21 | Acute systolic heart failure | Secondary outcome |
| Outcome definition | Secondary outcome | Acute/acute-on-chronic HF | ICD-10-CM | I50.23 | Acute on chronic systolic heart failure | Secondary outcome |
| Outcome definition | Secondary outcome | Acute/acute-on-chronic HF | ICD-10-CM | I50.31 | Acute diastolic heart failure | Secondary outcome |
| Outcome definition | Secondary outcome | Acute/acute-on-chronic HF | ICD-10-CM | I50.33 | Acute on chronic diastolic heart failure | Secondary outcome |
| Outcome definition | Secondary outcome | Acute myocardial infarction | ICD-10-CM | I21–I22 | Acute myocardial infarction | Secondary outcome |
| Outcome definition | Secondary outcome | Progression to advanced kidney disease | ICD-10-CM | Z99.2 N18.4 N18.5 N18.6 | Progression to advanced kidney disease | Secondary outcome |
| Outcome definition | Secondary outcome | All-cause death | Demographics | — | Deceased | Secondary outcome |

**Supplementary Table 2.** Comparison of hazard ratios (95% CIs) for clinical outcomes at 3 and 6 years before and after propensity score matching.

| Outcome | Before PSM  HR (95% CI) | After PSM  HR (95% CI) |
| --- | --- | --- |
| 3 YEARS |  |  |
| Composite | 1.61 (1.23–2.11) | 0.61 (0.40–0.93) |
| Acute/acute-on-chronic HF | 3.16 (2.20–4.55) | 0.99 (0.56–1.74) |
| Acute myocardial infarction | 2.13 (1.44–3.15) | 0.55 (0.28–1.05) |
| Progression to advanced kidney disease | 2.82 (1.97–4.03) | 1.06 (0.60–1.90) |
| All-cause death | 2.15 (1.65–2.80) | 0.61 (0.39–0.96) |
| 6 YEARS |  |  |
| Composite | 1.59 (1.27–1.99) | 0.65 (0.47–0.90) |
| Acute/acute-on-chronic HF | 2.90 (2.15–3.92) | 0.82 (0.54–1.26) |
| Acute myocardial infarction | 2.01 (1.45–2.79) | 0.55 (0.33–0.91) |
| Progression to advanced kidney disease | 3.07 (2.26–4.17) | 1.08 (0.68–1.73) |
| All-cause death | 1.88 (1.50–2.36) | 0.63 (0.43–0.93) |

**Supplementary Table 3.** Subgroup analyses of the 5-year composite outcome: hazard ratios before and after propensity score matching, and interaction p-values after matching.

| Subgroup | HR Before PSM (95% CI) | HR After PSM (95% CI) | P for interaction |
| --- | --- | --- | --- |
| Sex |  |  |  |
| Male | 1.28 (0.92–1.77) | 0.80 (0.50–1.26) |  |
| Female | 1.66 (1.16–2.36) | **0.40 (0.23–0.69)** | **0.14** |
| Age |  |  |  |
| ≤74 years | 2.08 (1.47–2.95) | 0.56 (0.30–1.05) |  |
| ≥75 years | 1.26 (0.90–1.75) | **0.53 (0.33–0.85)** | **0.92** |

**Supplementary Table 4.** Baseline characteristics before and after propensity score matching in the contemporary-period sensitivity analysis restricted to Fabry disease diagnoses recorded from 2013 onwards

|  | BEFORE | | | AFTER | | |
| --- | --- | --- | --- | --- | --- | --- |
| Variable | **Fabry**  **SGLT2i**  **exposed**  **(n=564)** | **Fabry**  **SGLT2i**  **non-exposed**  **(n=9,103)** | **Std Diff** | **Fabry**  **SGLT2i**  **exposed**  **(n=441)** | **Fabry**  **SGLT2i**  **non-exposed**  **(n=441)** | **Std Diff** |
| Age, years  (mean ± SD) | 65.3 ± 12.8 | 47.5 ± 24.3 | 0.921 | 64.7 ± 13.1 | 65.0 ± 12.9 | 0.017 |
| White, n (%) | 315 (55.9%) | 6,218 (68.3%) | 0.259 | 245 (55.6%) | 242 (54.9%) | 0.014 |
| Male, n (%) | 316 (56.0%) | 4,312 (47.4%) | 0.174 | 243 (55.1%) | 235 (53.3%) | 0.036 |
| Hypertension | 501 (88.8%) | 1,997 (21.9%) | 1.819 | 382 (86.6%) | 385 (87.3%) | 0.020 |
| Type 2 diabetes mellitus | 412 (73.0%) | 722 (7.9%) | 1.773 | 305 (69.2%) | 323 (73.2%) | 0.090 |
| Ischemic heart disease | 320 (56.7%) | 884 (9.7%) | 1.152 | 222 (50.3%) | 204 (46.3%) | 0.082 |
| Heart failure | 207 (36.7%) | 332 (3.6%) | 0.904 | 126 (28.6%) | 118 (26.8%) | 0.041 |
| Beta-blockers | 377 (66.8%) | 1,183 (13.0%) | 1.316 | 265 (60.1%) | 260 (59.0%) | 0.023 |
| ACE inhibitors | 264 (46.8%) | 762 (8.4%) | 0.953 | 197 (44.7%) | 207 (46.9%) | 0.046 |
| Angiotensin II receptor blockers | 279 (49.5%) | 518 (5.7%) | 1.124 | 193 (43.8%) | 183 (41.5%) | 0.046 |
| Aldosterone antagonists | 123 (21.8%) | 205 (2.3%) | 0.630 | 73 (16.6%) | 62 (14.1%) | 0.069 |
| Agalsidase beta | 48 (8.5%) | 108 (1.2%) | 0.346 | 27 (6.1%) | 23 (5.2%) | 0.039 |
| Agalsidase alfa | 19 (3.4%) | 22 (0.2%) | 0.237 | 11 (2.5%) | 12 (2.7%) | 0.014 |
| Pegunigalsidase alfa | 10 (1.8%) | 10 (0.1%) | 0.173 | 0 (0%) | 10 (2.3%) | 0.215 |
| Migalastat | 10 (1.8%) | 11 (0.1%) | 0.171 | 10 (2.3%) | 10 (2.3%) | <0.001 |
| eGFR, mL/min/1.73 m² (mean ± SD) | 72.9 ± 26.8 | 87.8 ± 23.9 | 0.585 | 75.5 ± 26.1 | 79.3 ± 22.5 | 0.156 |

***Footnote****: PSM, propensity score matching; SD, standard deviation; eGFR, estimated glomerular filtration rate; SGLT2i, sodium–glucose cotransporter-2 inhibitor. Standardized differences <0.1 indicate adequate balance between groups.*

**Supplementary Table 5.** Baseline characteristics in the sensitivity analysis requiring ≥2 Fabry disease diagnostic records

|  | BEFORE | | | AFTER | | |
| --- | --- | --- | --- | --- | --- | --- |
| Variable | **Fabry**  **SGLT2i**  **exposed**  **(n=550)** | **Fabry**  **SGLT2i**  **non-exposed**  **(n=6,104)** | **SMD** | **Fabry**  **SGLT2i**  **exposed**  **(n=299)** | **Fabry**  **SGLT2i**  **non-exposed**  **(n=299)** | **SMD** |
| Age at index, years | 66.2 ± 12.2 | 46.6 ± 23.3 | 1.0600 | 63.4 ± 12.4 | 63.7 ± 11.4 | 0.0281 |
| White race | 322 (58.5) | 4,075 (66.8) | 0.1704 | 171 (57.2) | 169 (56.5) | 0.0135 |
| Male sex | 312 (56.7) | 2,842 (46.6) | 0.2045 | 155 (51.8) | 161 (53.8) | 0.0402 |
| Hypertensive diseases | 477 (86.7) | 1,031 (16.9) | 1.9540 | 243 (81.3) | 247 (82.6) | 0.0348 |
| Type 2 diabetes mellitus | 397 (72.2) | 442 (7.2) | 1.7742 | 200 (66.9) | 212 (70.9) | 0.0868 |
| Ischaemic heart disease | 274 (49.8) | 393 (6.4) | 1.1014 | 115 (38.5) | 108 (36.1) | 0.0484 |
| Heart failure | 178 (32.4) | 147 (2.4) | 0.8604 | 66 (22.1) | 60 (20.1) | 0.0492 |
| Beta-blocking agents | 366 (66.5) | 552 (9.0) | 1.4728 | 161 (53.8) | 157 (52.5) | 0.0268 |
| ACE inhibitors | 287 (52.2) | 383 (6.3) | 1.1692 | 126 (42.1) | 130 (43.5) | 0.0270 |
| Angiotensin II receptor blockers | 277 (50.4) | 281 (4.6) | 1.1937 | 113 (37.8) | 102 (34.1) | 0.0767 |
| Aldosterone antagonists | 121 (22.0) | 113 (1.9) | 0.6541 | 42 (14.0) | 41 (13.7) | 0.0097 |
| Agalsidase beta | 56 (10.2) | 38 (0.6) | 0.4326 | 13 (4.3) | 10 (3.3) | 0.0522 |
| Agalsidase alfa | 19 (3.5) | 0 (0.0) | 0.2675 | 10 (3.3) | 0 (0.0) | 0.2631 |
| Pegunigalsidase alfa | 10 (1.8) | 0 (0.0) | 0.1925 | 10 (3.3) | 0 (0.0) | 0.2631 |
| Migalastat | 15 (2.7) | 10 (0.2) | 0.2160 | 10 (3.3) | 10 (3.3) | <0.0001 |
| eGFR, mL/min/1.73 m²  (mean ± SD) | 72.9 ± 25.6 | 88.0 ± 22.9 | 0.6235 | 78.2 ± 24.7 | 78.6 ± 20.9 | 0.0159 |

**Supplementary Table 6. Outcomes in sensitivity analysis**

|  | BEFORE | | | | AFTER | | | |
| --- | --- | --- | --- | --- | --- | --- | --- | --- |
| Outcome | **Fabry SGLT2i**  **exposed, (events/N (%))** | **Fabry SGLT2i non-exposed**  **events/N (%)** | **HR**  **(95% CI)** | **P value** | **Fabry SGLT2i-exposed, events/N (%)** | **Fabry SGLT2i non-exposed, events/N (%)** | **HR (95% CI)** | **P value** |
| Primary composite outcome | 40/429 (9.32) | 577/6,060 (9.52) | 1.43  (1.04–1.97) | 0.0278 | 14/248 (5.65) | 39/260 (15.00) | 0.51  (0.28–0.94) | 0.0275 |
| Acute or acute-on-chronic heart failure | 24/476 (5.04) | 170/6,130 (2.77) | 2.67  (1.74–4.11) | <0.0001 | NR | NR | NE | — |
| Acute myocardial infarction | 19/472 (4.03) | 216/6,089 (3.55) | 1.68  (1.05–2.69) | 0.0283 | NR | NR | NE | — |
| Progression to advanced kidney disease | 35/498 (7.03) | 176/6,081 (2.89) | 3.38  (2.35–4.87) | <0.0001 | 20/281 (7.12) | 17/289 (5.88) | 1.72  (0.90–3.29) | 0.0999 |

***Footnote:*** *The primary composite outcome comprised acute myocardial infarction, all-cause mortality, and acute or acute-on-chronic heart failure. Patients with the corresponding outcome before the follow-up window were excluded; therefore, denominators differ across outcomes. HRs and p values were derived from Kaplan–Meier analyses. NR, not reported because the event count was below the minimum TriNetX reporting threshold; NE, not estimable.*

**Supplementary Figure 1**. Love plot showing covariate balance before and after propensity score matching.

***Footnote:*** *Absolute standardized mean differences (SMDs) are shown for all covariates included in the propensity score matching procedure before (red diamonds) and after (blue circles) matching. The vertical dashed line indicates the conventional threshold for adequate covariate balance (SMD = 0.10). Lower SMD values indicate better balance between the SGLT2 inhibitor-exposed and non-exposed cohorts.*

**Supplementary Figure 2**. Forest plot of outcomes 3 years and 6 years before and after propensity score matching (PSM).

***Footnote****: HF indicates heart failure*

**Supplementary Figure 3**. Five-year clinical outcomes in the contemporary-period sensitivity analysis

***Footnote:*** *Forest plots show hazard ratios and 95% confidence intervals for the study outcomes before (Panel A) and after (Panel B) propensity score matching. Hazard ratios below 1 favour the SGLT2i-exposed cohort. The hazard ratio for acute myocardial infarction after propensity score matching could not be estimated because of the insufficient number of events. The dashed vertical line indicates a hazard ratio of 1. CI, confidence interval; FD, Fabry disease; HF, heart failure; HR, hazard ratio; PSM, propensity score matching; SGLT2i, sodium–glucose cotransporter-2 inhibitor.*
